# Comparison of Various Minimally Invasive Techniques for the Management of Gingival Hyperpigmentation: A Case Report

**DOI:** 10.7759/cureus.73870

**Published:** 2024-11-17

**Authors:** Lekha Ashokkumar, Dhanadivya Krishnakumar, Deepak M Ravindran, Surbhi Jain, Divya Kumar, Balaji SK

**Affiliations:** 1 Department of Periodontology, Sri Ramachandra Dental College and Hospital, Sri Ramachandra Institute of Higher Education and Research, Chennai, IND

**Keywords:** depigmentation of gingiva, gingival hyperpigmentation, laser dentistry, minimally invasive dentistry, oral mesotherapy, smile esthetics

## Abstract

Gingival hyperpigmentation is a condition wherein there is excessive deposition of melanin pigment, which is produced by the melanocytes of the gingiva. Gingival depigmentation is a periodontal surgical procedure whereby the pigmentation is removed or reduced by various surgical techniques that are associated with significant postoperative pain, bleeding, and recurrence. Laser ablation has been recognized as one of the minimally invasive, effective, comfortable, and reliable techniques for gingival depigmentation. The use of lasers has several advantages such as high patient compliance, short healing period, no or very slight pain, and hemorrhage. On the contrary, mesotherapy is a minimally invasive technique that uses fine-gauge needles to locally administer modest amounts of Vitamin C to the mesoderm layer to achieve cosmetic goals. Recently, some case studies have shown that ascorbic acid (Vitamin C) can be used in the management of gingival pigmentation. The objective of this case report is to compare the clinical efficacy of these two techniques for gingival depigmentation and to arrive at an effective minimally invasive alternative for gingival depigmentation.

## Introduction

A smile is often considered a beautiful and appealing expression. The perfect proportion and harmonious combination of pink and white aesthetics play an important role in enhancing the overall smile and appearance.

Gingival pigmentation is a common condition in which the gingiva appears darker due to the presence of melanin produced by specialized cells called melanocytes. The amount of melanin present in the gingiva varies in individuals and can be a source of aesthetic concern for some [1]. Gingival depigmentation is a procedure aimed at removing melanin from the gingival tissue to enhance the aesthetic appearance. Traditionally, this condition has been addressed through conventional surgical techniques, which often entail considerable discomfort and extended recovery times. Recent advancements in dental aesthetics have introduced less invasive methods, notably the use of lasers and vitamin C, both of which have shown effective results in improving gum health and appearance with minimal discomfort [2].

Laser is a promising treatment modality for dental procedures, which offers precision and efficacy, allowing for controlled ablation of pigmented tissues while minimizing damage to surrounding healthy gingiva [3]. Vitamin C, known for its potent antioxidant properties, plays an important role in collagen synthesis and tissue repair. The integration of oral mesotherapy with biocompatible agents like vitamin C serves as a novel and less invasive treatment alternative with favorable outcomes [4].

Mesotherapy is a minimally invasive technique that involves the injection of small amounts of therapeutic agents into the mesoderm, the middle layer of skin, to promote healing and rejuvenation [5]. Vitamin C is well-known for its antioxidant capacity, promoting collagen synthesis and enhancing the overall health of gum tissues. When utilized for gingival depigmentation, mesotherapy methods can distribute vitamin C evenly into the affected areas, harnessing its properties to break down melanin while also facilitating tissue repair and renewal. Vitamin C interacts with copper and scavenges reactive oxygen species, thereby inhibiting the tyrosinase enzyme activity leading to reduced melanin synthesis [6].

Therefore, this article aims to compare and assess the clinical effectiveness and patient-reported outcomes of intraepidermal (oral mesotherapy) vitamin C injection and lasers for nonsurgical management of physiologic gingival melanin hyperpigmentation. By analyzing the outcomes associated with this approach, we seek to assess its viability and feasibility as a standard practice in the periodontal treatment of gingival depigmentation.

## Case presentation

Case 1

A 20-year-old female patient reported to the outpatient Department of Periodontics, Sri Ramachandra Dental College and Hospital, SRIHER, with a chief complaint of dark spots on her gums, which was unpleasant while smiling. The patient was systemically healthy and intraoral examination revealed diffuse moderate gingival pigmentation in the maxillary and mandibular gingiva. The patient was explained about the minimally invasive gingival depigmentation procedure using vitamin C and a 940 nm diode laser. An informed consent was obtained from the patient prior to the procedure. The patient’s oral hygiene maintenance was good and Dummet’s oral pigmentation index, 1964 (DOPI) score was 2, suggesting moderate pigmentation [7]. Full mouth scaling was performed and oral hygiene instructions were given. Intraoral photographs were taken prior to the depigmentation procedure. Local anesthetic infiltration was given in the first quadrant (11 to 15 region - FDI tooth numbering system). A 940 nm diode laser with a fiber optic tip diameter of 400 µm was used at 1 W power. Laser ablation of the gingiva was performed with a fiberoptic tip in contact mode, using a continuous light feather stroke extending from the midline to the distal area, between the muco-gingival junction and the gingival margin, including the interdental papilla.

**Figure 1:**
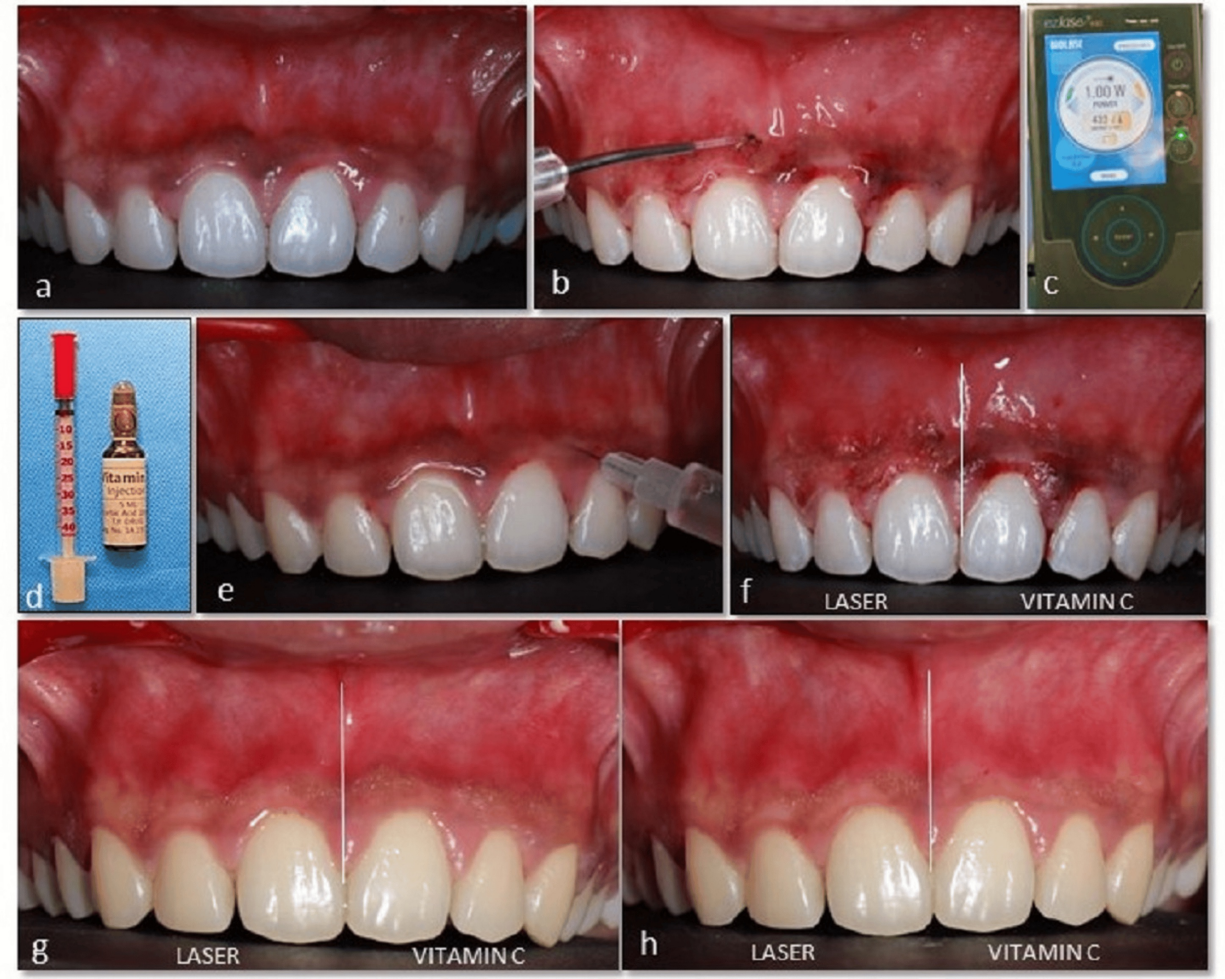
Sequence of gingival depigmentation using laser and vitamin C. (a) Pre-operative image. (b,c) De-epithelialization using a 940 nm diode laser in the first quadrant. (d) Insulin administering syringe and vitamin C. (e) Intra-epithelial injection of vitamin C in the second quadrant. (f) Immediate post-operative image. (g) One-month follow-up. (h) Six-month follow-up.

The local anesthetic spray was applied in the second quadrant (21 to 25 region - FDI tooth numbering system). Intra-epithelial injection of vitamin C at a concentration of 250 mg (Vesco Pharmaceutical, Bangkok, Thailand) was administered using an insulin syringe with a 30-gauge needle. The needle was inserted at a depth of 0.5 mm to 1.0 mm at points spaced 2 mm to 3 mm apart. 0.1 mL of vitamin C was deposited at each point until blanching of the gingiva was observed. The same procedure was repeated once every week for four weeks [8,9]. Post-operative instructions were given and the patient was advised to refrain from having citrus, spicy foods, or foods with color additives. Visual analog scale (VAS) assessing the pain was recorded at one week post-treatment and the gingival pigmentation was reassessed at one, three, and six months post-operatively (Figure 1).

Case 2

**Figure 2:**
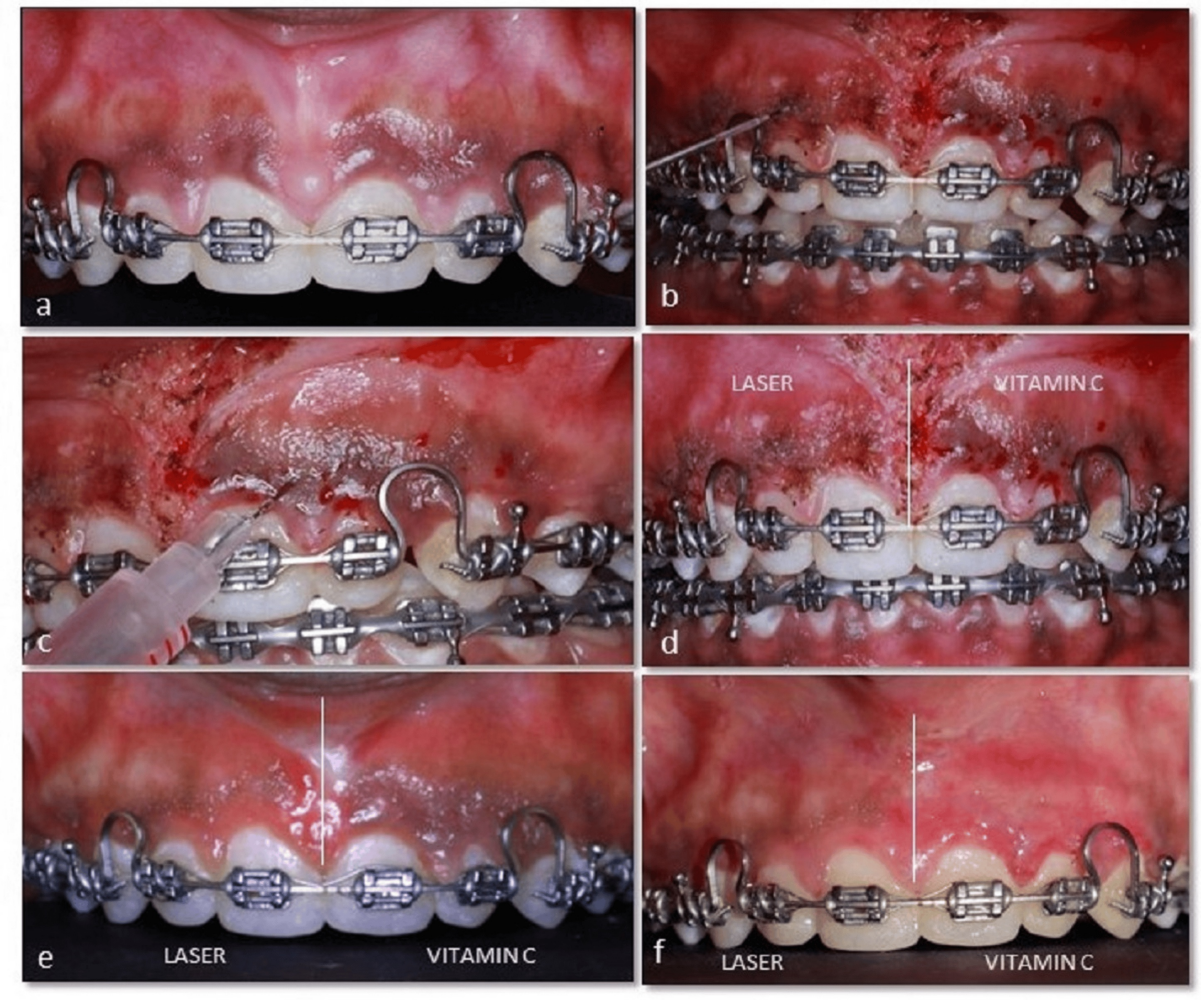
Sequence of gingival depigmentation using laser and vitamin C. (a) Pre-operative image. (b) De-epithelialization using a 940 nm diode laser in the first quadrant. (c) Intra-epithelial injection of vitamin C in the second quadrant. (d) Immediate post-operative image. (e) One-month follow-up. (f) Six-month follow-up.

A 21-year-old female patient reported to the outpatient Department of Periodontology, Sri Ramachandra Dental College and Hospital, SRIHER, with a chief complaint of discoloration of her gums and unaesthetic smile. The patient had no relevant medical history and was under fixed orthodontic treatment for the past year. Intra-oral examination revealed high frenum attachment in relation to 11, 21 and diffuse moderate gingival pigmentation (DOPI score: 2). Frenectomy was done using a 940 nm diode laser at 1 W power in continuous mode. Gingival depigmentation was done using a diode laser in the first quadrant (11 to 25 region - FDI tooth numbering system) and intraepithelial injection of vitamin C in the second quadrant (21 to 25 region - FDI tooth numbering system) as described earlier. The clinical parameters were assessed at one, three, and six months postoperatively (Figure 2).

Clinical and patient-related outcome

The gingival pigmentation at one, three, and six months after therapy was assessed using the Dummett-Gupta oral pigmentation index, 1964 (DOPI) and the Gingival pigmentation index (GPI) [7,10]. There was a significant improvement in DOPI and GPI scores in both treatment sites at one-month follow-up. There was no significant change in gingival color at the three-month and six-month follow-ups.

The intensity of pain was assessed using VAS, one week after the depigmentation procedure, and improvement in gingival color as observed by the patient was assessed on a scale of 0-4 at one, three, and six months after treatment [9,11,12].

## Discussion

A comparative analysis of clinical and patient-related outcomes following laser-assisted gingival depigmentation and intraepithelial injection of vitamin C reveals beneficial results. The improvement in gingival color in the vitamin C quadrant was equally effective as laser-assisted gingival depigmentation in both cases. The pigmentation intensified immediately after administering vitamin C injection due to its interaction with melanin-containing cells causing the release of pigments [13].

The DOPI score significantly improved at one month when compared to the baseline suggesting no clinical evidence of pigmentation in both cases. During the follow-up visits, a gradual improvement in the gingival pigmentation was noted, and a homogenous pink color was observed during the one-month follow-up, which was similar to the laser-assisted depigmentation sextant. No signs of re-pigmentation were observed in both quadrants during the three- and six-month follow-ups, thus showing the consistency of the new treatment strategy.

Patient-related outcomes assessed during the first-week recall visit showed encouraging results with mild pain (VAS score: 1) perceived by both the patients in the laser-treated sextant and no pain (VAS score: 0) in the vitamin C sextant.

A systematic review and meta-analysis by Meisha et al. in 2019 suggest that laser-assisted gingival depigmentation had better esthetic outcomes and patient satisfaction when compared to the conventional method [14]. Ahoud and Hebab in their recent systematic review and meta-analysis in 2023 suggested it as highly technique-sensitive and requires sophisticated instruments [2].

Vitamin C as a depigmentation agent has shown promising results. The topical application of ascorbic acid gel was found to be effective in treating gingival pigmentation [15]. Sheel et al. in 2015 showed the absence of recurrence of pigmentation at nine months following surgical depigmentation with adjunctive application of topical vitamin C gel [16]. Brahmavar et al. and Yussif et al. in their series of clinical studies have proved oral mesotherapy with vitamin C significantly reduced the gingival pigmentation when compared to the gold standard scalpel technique [11,17,18]. A recent study by El-Mofty et al. in 2021 showed that vitamin C when given as an intraepithelial injection showed better and early effects when compared to topical application and it was also proved to be less painful and esthetically satisfying [19]. In conjunction with the previous studies, our case report has also shown promising clinical outcomes, esthetic satisfaction, less pain, and improved patient satisfaction when treated with vitamin C injections. The primary limitation of using vitamin C as an oral mesotherapy agent is the requirement for multiple visits and at least four weeks for a homogeneous color to appear. To the best of our knowledge, this is the first split-mouth study to compare the clinical efficacy of laser-assisted gingival depigmentation with oral mesotherapy using vitamin C in the management of gingival hyperpigmentation.

## Conclusions

Vitamin C as oral mesotherapy is an effective and safe approach for managing gingival hyperpigmentation. Although diode laser yields better and earlier results, oral mesotherapy with vitamin C offers a minimally invasive and applicable treatment option that is both cost-effective and esthetically satisfying for the patients. Furthermore, to assess the longevity of the effectiveness of vitamin C as an intraepithelial injection, randomized clinical trials with a larger sample size are required to suggest it as an effective minimally invasive treatment alternative for the management of gingival hyperpigmentation. 
